# Unique Presentation of Akinetic Mutism and Coexisting Thyroid Storm Relating to Stroke

**DOI:** 10.1155/2014/320565

**Published:** 2014-10-28

**Authors:** Mohankumar Kurukumbi, Thao Dang, Najeeb Crossley, Alice Esame, Annapurni Jayam-Trouth

**Affiliations:** ^1^Department of Neurology, Howard University Hospital, 2041 Georgia Avenue, Washington, DC 20060, USA; ^2^Department of Neurology, Howard University College of Medicine, Washington, DC 20060, USA

## Abstract

Akinetic mutism is described in various clinical presentations but typically is defined as a state wherein the patient appears awake but does not move or speak. It can be divided into two different subtypes; the most common subtypes depend on the lesion location, mesencephalic-diencephalic region, also called apathetic akinetic mutism (somnolent mutism), and those involving the anterior cingulate gyrus and adjacent frontal lobes called hyperpathic akinetic mutism. The pathway of akinetic mutism is believed to originate from circuits that link the frontal and subcortical structures. This case reports a 48-year-old African American female with bilateral anterior cerebral artery stroke and akinetic mutism with coexisting thyroid storm. This patient with bilateral anterior cerebral artery infarcts presented with characteristics that are typical for akinetic mutism such as having intact eye movements but an inability to respond to auditory or visual commands. With the incidence of bilateral anterior cerebral artery (ACA) ischemic stroke being rare and the incidence of akinetic mutism secondary to ischemic stroke even rarer, we suspect that this patient potentially had a unilateral occlusion of anomalous anterior cerebral vasculature.

## 1. Introduction

The incidence of bilateral anterior cerebral artery (ACA) ischemic stroke is rare and the incidence of akinetic mutism secondary to ischemic stroke is even rarer. Akinetic mutism is a condition described typically as a state wherein a patient appears awake but cannot move or speak. This case report describes a 48-year-old African American female with bilateral ACA stroke and akinetic mutism with coexisting thyroid storm.

## 2. Case Presentation

The patient is a 48-year-old African American female with a past medical history of hypertension, hyperthyroidism, diabetes mellitus, chronic knee, and back pain who presented to the hospital in an aphasic state after being found unresponsive by her son. Emergency care area evaluation showed right-sided weakness and questionable left leg weakness with an NIH stroke assessment score of 5/42. The patient was admitted and managed under acute stroke protocol.

**Figure 1:**
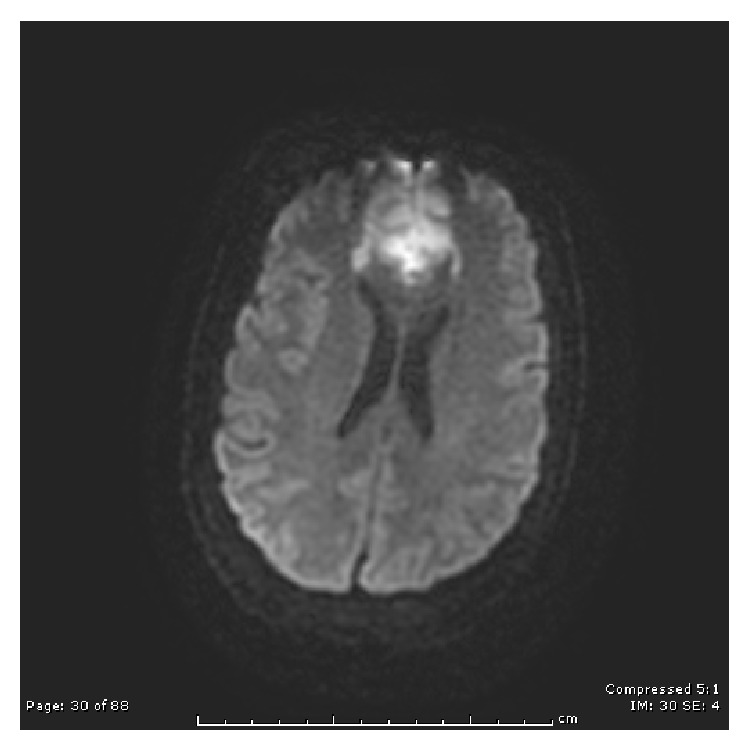
DWI MRI showing restricted diffusion bilateral ACA territory.

**Figure 2:**
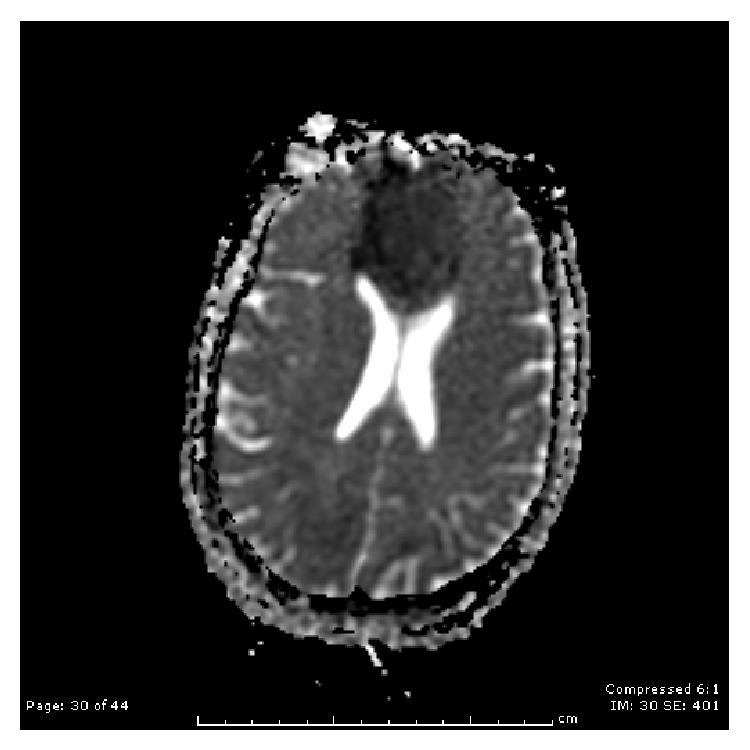
ADC MRI showing mismatch bilateral ACA territory.

**Figure 3:**
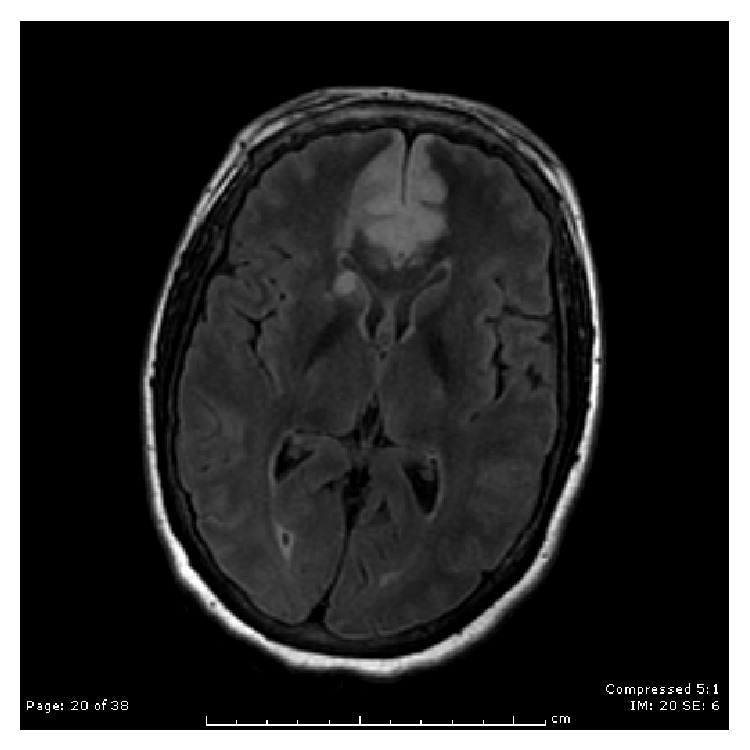
FLAIR MRI showing hyperintense lesion bilateral ACA territory and head of caudate on right side.

The results of a brain magnetic resonance imaging (MRI) scan without contrast showed large bilateral ACA infarcts involving the entire vascular territory, an acute caudate head infarct, severe stenosis in the proximal right M1 segment, and thyroid enlargement (Figures 1, 2, and 3). Upon examination, the patient was noted to be unresponsive to visual, auditory, and verbal commands but noted to be alert. Although not responding by emotional or motor movements, ocular movements and the ability to follow visual stimuli were intact. She was diagnosed with akinetic mutism on the basis of the imaging results and the physical exam and a regimen of bromocriptine was started. The patient responded positively to the treatment for about a week after which her condition began to deteriorate.

Note that all other possible confounding factors for stroke were excluded by doing additional tests, which are all normal, including hemoglobin electrophoresis, paroxysmal nocturnal hemoglobinuria marker, proteins S and C, antithrombin III, factor Leiden V, erythrocyte sedimentation rate, antinuclear antibody, rapid plasma reagin, anticardiolipin antibody, vitamin B12 levels, homocysteine levels, prothrombin and partial prothrombin times, hemoccult stool, TEE, and lupus anticoagulant antibodies. EKG showed sinus tachycardia and no ischemic or arrhythmic changes were detected.

The patient was also found to have hyperthyroidism at admission (free T4: 6.15, TSH: 0.01) and based on these values and recommendations from the endocrine team, the patient was thought to have thyroid storm and transferred to the medical intensive care unit. Patient was started on propylthiouracil, propranolol, hydrocortisone, and potassium iodide. Over a period of 14 days, the patient's hyperthyroidism was noted to be resolving with normalization of thyroid function tests. Of note, this patient has a history of long standing hyperthyroidism on regular thyroid supplementation. This patient has been examined for antithyroglobulin antibody and antithyroid peroxidase antibodies in the past as well as the current admission, and the results were negative.

Her hospital course was further complicated by urosepsis, which was treated, and hemorrhagic conversion of the right anterior cerebral artery (ACA) infarct. She was maintained on hyperthyroidism treatment and continued on secondary stroke preventions. Gradually, patient improved and was transferred to a long term rehabilitation center.

## 3. Discussion

Infarction of the anterior cerebral artery accounts for 1% to 4.4% of cerebral infarctions [1, 2]. This small percentage is due to collateral circulation provided by the anterior communicating arteries. Bilateral anterior cerebral artery infarction in a young patient such as ours is extremely rare. We suspect that this patient potentially had a unilateral occlusion of the anomalous anterior cerebral vasculature arising from the patient's right internal carotid artery.

**Figure 4:**
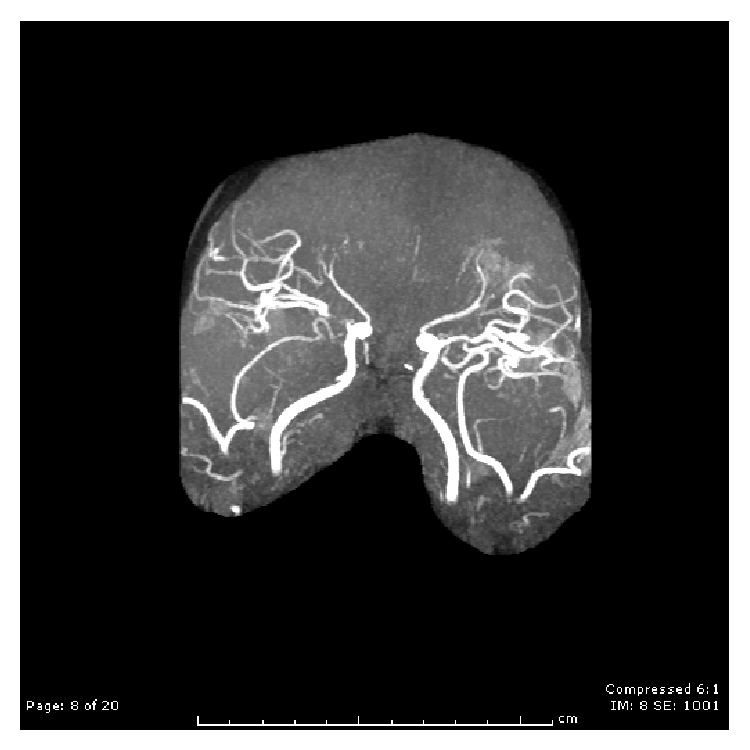
MRA showing pruning of bilateral ACA territory.

Anterior cerebral arteries (ACA) supply the medial aspect of the cerebral hemispheres. They are divided into A1, A2, and A3 segments. The most frequently observed variation is unilateral aplasia of A1 segment. At the A2 segment, there are 3 types of variations, which include bihemispheric (asymmetric) ACA, triple ACA (presence of the median artery of corpus callosum), and unpaired (azygos) ACA [3]. Imaging could not confirm the presence of an anomalous ACA in this patient because of the severity of the occluded vessel and timing of the infarct. However, based on literature and the patient's clinical presentation, it is suspected that she has an anomalous anterior cerebral vasculature resulting in simultaneous bilateral occlusion of ACA due to thrombosis or embolism, which subsequently contributed to her diagnosis of akinetic mutism (Figure 4).

Akinetic mutism is described in various clinical presentations but typically is defined as a state wherein the patient appears awake but does not move or speak. This patient with bilateral anterior cerebral artery infarct presented with similar characteristics such as having intact eye movements while being unable to respond to auditory or visual commands. Freemon described a patient with a bilateral ACA infarct as exhibiting behavior of reduced activity or slowness, marked speech reduction, and decreased facial expression and gestures [4].

Nagaratnam et al. noted that akinetic mutism can be divided into two different subtypes; the most common subtypes depend on the lesion location, mesencephalic-diencephalic region, also called apathetic AM (somnolent mutism), and those involving the anterior cingulate gyrus and adjacent frontal lobes called hyperpathic AM [5]. Hyperpathic AM produces a more severe presentation because it is associated with the bilateral cingulate gyrus in the territory of the ACA. The pathway of akinetic mutism is believed to originate from circuits that link the frontal and subcortical structures. While as many as five circuits are believed to be involved, it is postulated that the circuit involved in this patient's presentation involves anterior cingulate circuit. This circuit originates from the anterior cingulate gyrus, projecting to the ventral striatum to the ventral and rostrolateral globus pallidus and rostrodorsal substantial niagra, which then projects to the paramedian part of the medial dorsal nucleus of the thalamus and finally back to the anterior cingulate cortex. Its pathology has been explained by damage to this region that is responsible for decreased motivation and akinetic mutism. As for the caudate nucleus infarct and its relationship to akinetic mutism, it is possible that the bilateral ACA ischemic stroke could impede the striatal efferent projections from the caudate nucleus, thus leading to behavior abnormalities seen in akinetic mutism.

Etiologies for akinetic mutism are seen not only in our patient's condition but also in other conditions such as meningitis, encephalitis, hydrocephalus, trauma, tumors, and third ventricular cysts to vascular lesions like ruptured aneurysm and infarction [5]. According to Nagaratnam et al., akinetic mutism may also be complicated by certain illnesses such as psychiatric disorders, including catatonic schizophrenia, severe depression, or conversion reaction. Often, it may also be misdiagnosed as delirium, depression, or locked-in-syndrome since these conditions are also commonly associated with stroke.

Dopamine agonists and levodopa have commonly been used in the treatment of akinetic mutism; thus bromocriptine was initiated in our patient. It has been postulated that the underlying mechanism of akinetic mutism is injury to dopaminergic pathways and neurons; however this has not been proven. Yang et al. tried to demonstrate this causal relationship by using SPECT imaging to examine the presynaptic dopamine receptors in a patient with akinetic mutism. Images taken showed damaged dopamine receptors when the patient was symptomatic and restoration of the receptors when the patient was asymptomatic [6].

Strong research regarding the relationship between hyperthyroidism/thyroid storm and stroke is sparse. Case reports and a few cohort studies comprise the majority of the available literature. In Taiwan a prospective study compared 3176 hyperthyroid patients between 18 and 44 years of age with appropriately matched controls for 5 years after initial diagnosis and found that the hazard of having ischemic stroke during that time period was 1.44 times greater (95% CI, 1.02–2.12; *P* = 0.038) for patients with hyperthyroidism than for patients in the comparison cohort. An isolated case report of a 62-year-old female linked thyroid storm and subsequent atrial fibrillation with ischemic stroke secondary to poor INR control from anticoagulation medication. In our patient an underlying paroxysmal a-fib cannot be excluded.

## 4. Conclusion

The occurrence of bilateral ACA territory stroke is very rare. Occurrence of these three entities together, bilateral ACA territory stroke, akinetic mutism, and coexisting thyroid storm, is an even rarer combination and has never been reported in the literature.

## Abbreviations

ACA: Anterior cerebral artery
AM: Akinetic mutism
ECA: Emergency care area
MRI: Magnetic resonance imaging
SPECT: Single-photon emission computed tomography
TEE: Transesophageal echocardiogram.
